# Variations in isochore thickness and depositional surface of the Dwyka, Ecca and Beaufort Groups in the Western Cape Province of South Africa as deduced from 2.5D gravity profile models

**DOI:** 10.1016/j.heliyon.2021.e06478

**Published:** 2021-03-12

**Authors:** Zusakhe Nxantsiya, Oswald Gwavava, Christopher Baiyegunhi

**Affiliations:** a Council for Geoscience, Private Bag X112, Pretoria, 0001, Gauteng Province, South Africa; b Department of Geology, Faculty of Science and Agriculture, University of Fort Hare, Private Bag X1314, Alice, 5700, Eastern Cape Province, South Africa; c Department of Geology and Mining, School of Physical and Mineral Sciences, University of Limpopo, Private Bag X1106, Sovenga, 0727, Limpopo Province, South Africa

**Keywords:** Gravity modelling, Isochore thickness, Depositional surface, Karoo basin

## Abstract

A 2.5D gravity modelling along seven selected profiles that covers the Western Cape Province of South Africa was carried out to deduce the depositional surface and isochore (true vertical) thickness of the Dwyka, Ecca and Beaufort Group sediments. The results revealed that the Karoo Basin deepens to a depth of about 4600 m in the south-western region, near the front of the Cape Fold Belt. Also, the model gives indication that the Karoo dolerite intrusions are interconnected at depth and are mostly concentrated at the centre of the basin. The isochore thickness maps show that the Beaufort Group is the thickest group in the Karoo Supergroup, with a maximum thickness of about 6046±277 m, followed by the Ecca and Dwyka Groups with thicknesses of around 3720±183 m and765±69 m, respectively. The maximum depositional surface (elevation) for the Dwyka, Ecca and Beaufort sediments are approximately 1696 m, 1247 m and 1491 m, respectively, whereas the maximum depth below sea level are around 3668 m, 3209 m and 480 m, respectively. Furthermore, the isochore thickness maps indicate that the Ecca Group, which is the main target for hydrocarbon exploration in the Karoo, thickens toward the south, away from the centre of the basin and reaches thickness of greater than 3680 m. The correlation of the depositional surfaces with the isochore thickness maps revealed that the sediments in structural high areas were subsided, eroded and deposited in structural low areas. Consequently, the structural low areas are characterised by thick sediments cover and vice versa.

## 1 Introduction

**Figure 1:**
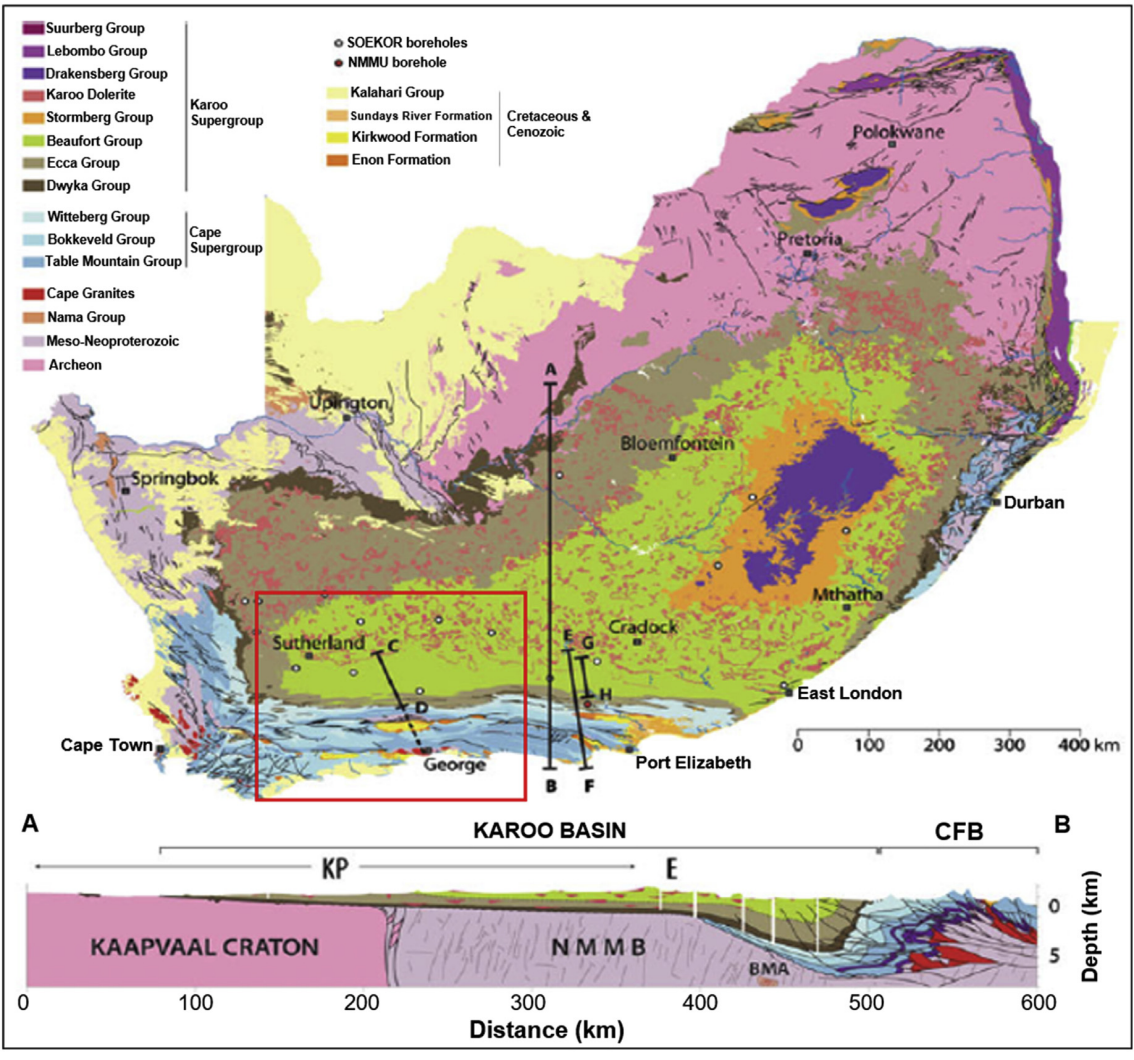
Geological map of South Africa as compiled by the Council for Geosciences showing distributions of lithostratigraphic units and the location of geophysical profiles in the Karoo Basin. Note: The red square indicate the boundaries of the study area.

The Karoo Basin, particularly the area under investigation, is of interest to scientists and resource economists. From the perspective of the economists, shale gas reserves of the Karoo Basin in South Africa have been estimated to have a potential to positively influence the economy of the country. The south-western Karoo Basin is considered to be one of the prospective areas for shale gas in South Africa due to the presence of deeply buried, thermally mature black shales (Baiyegunhi et al., 2017). The study area covers part of the south-western Karoo and Cape Supergroups in the Western Cape Province of South Africa. Geographically, the area extends from longitudes 20**°** E to 24**°** E and latitudes -31**°** S to -34**°** 50’ S (Figure 1). The Cape Fold Belt comprises of the Palaeozoic clastic sedimentary rocks of the Cape Supergroup that rests unconformably on the Precambrian basement rocks (Bachtadse et al., 1987) and are in turn overlain by the rocks of the Karoo Supergroup (Tankard et al., 2012). The Cape Supergroup is reported to have been deposited during the rising and falling of the ocean along a shallow continental-shelf that flanks the southern margin of Gondwana (Linol et al., 2016).

Geological interpretation of the evolution of the Karoo Basin vary from intracratonic thermal sag basin fills (Bordy, 2000), to a retro-arc foreland basin that developed due to shallow angle subduction of the palaeo-Pacific plate underneath the Gondwana supercontinent (Catuneanu et al., 2002; Catuneanu, 2004; Johnson et al., 2006), a transtensional foreland basin formed as a result of subsidence and tilting in a strike-slip regime (Tankard et al., 2009, 2012), to a thin-skinned fold belt that formed as a result of collisional tectonics and distant subduction (Lindeque et al., 2011; Pangaro and Ramos, 2012). The basin forms the thickest and most complete stratigraphic sequence of several depositories of the Permo-Carboniferous to Jurassic age in the south-western Gondwana (Catuneanu and Elango, 2001). This sedimentary sequence reaches a maximum thickness of about 5 km in front of the Cape Fold Belt and reflects a variety of palaeoclimatic conditions and depositional environments, varying from glacial to marine, deltaic, fluvial and aeolian (Smith et al., 1993, Figure 1). These palaeoenvironments have been described by Linol et al. (2016) to reflect a special time in the history of Earth during which the supercontinent Gondwana amalgamated and broke-up (750-130 Ma).

Tectonism and climate are the two main allogenic controls that influenced the sedimentary fill of the Karoo Basin (Catuneanu et al., 1998, 2005). During the deposition of the Karoo Supergroup in Late Carboniferous, the tectonic regimes changed from mainly flexural in the south, in response to processes of subduction, accretion and mountain building along the Panthalassan (palaeo-Pacific) margin of Gondwana, to extensional in the north, in response to processes of spreading along the Tethyan margin of Gondwana (Tankard et al., 2012). According to Catuneanu et al. (2005), the tensional episodes that were initiated during the Karoo time led to the development of the early Tethyan spreading centre and controlled deposition in the Karoo Basin until the break-up of Gondwana in the Middle Jurassic. Propagation of tensional stresses from the Tethyan margin to the south started during the Late Carboniferous, thus controlling the rate of deposition of Karoo sediments in the already developed grabens and rift structures. The factors that affect the type, distributions and amount of sediments in the structural or basement low areas with respect to time are best explained when the sedimentation rates as well as the horizontal and vertical changes are known. This is a function of the initial structural control of the depositional surface/or nature of the underlying basement prior to deposition (James et al., 2003).

Generally, the structural low areas are mostly associated with depositional processes, resulting in thick sediment cover, whereas structural high areas usually experience removal processes like weathering and erosion, thus causing thin sediment layers. As documented by Zhou et al. (2015), sediment thickness in structural lows and high areas are mostly a direct result or configuration of the underlying basement or crustal topography. This direct relationship could be possibly linked to erosion of sediments from structural highs, and subsequent transportation and deposition of the sediments in structural low areas (Chen et al., 2015). The Karoo retro-arc foreland system formed through flexural deflection of the lithosphere in relation to a mixture of supra- and sublithospheric loads (Catuneanu et al., 1998). The changes or variations in accommodation within the Karoo Basin were modelled by Catuneanu et al. (1998) and they pointed out that the accommodation is controlled by the flexural response of the lithosphere to orogenic cycles or episode of loading and unloading. The supracrustal loading orogens led to the subdivision of the Karoo foreland system into flexural provinces (i.e. foredeep and forebulge). The subsequent addition of loads into the orogenic belts by thrusting, resulted in subsidence of the foredeep and uplift of the forebulge, but opposite occurred when orogenic load was removed by extension or erosion. This trend of opposite or reverse vertical tectonics alters the comparative quantity or thickness of available accommodation in the two flexural provinces (foredeep and forebulge) and may create out of phase proximal to distal stratigraphies (Catuneanu, 2004).

The inter-relationship between the base level changes and supply of sediment controls the extent in which the available accommodation was used up by sedimentation (Miall, 1997). Despite over 30 years research in the main Karoo Basin of South Africa, the knowledge of variation in sediment thicknesses over the Karoo remains poorly documented. The results of the deep boreholes drilled by the Southern Oil Exploration Corporation (SOEKOR) in the study area revealed the thicknesses of various groups within the boreholes. However, these thicknesses vary across the study area as a result of the Cape deformation. Consequently, it is necessary to determine the variation in isochore thicknesses of the geologic groups in order to ascertain the thickness of the sediments across the study area. Based on the gravity profile modelling, this paper presents the depositional surfaces (elevation) and isochore (true vertical) thickness maps of the Dwyka, Ecca and Beaufort Groups across the study area. Also, the depositional surfaces and isochore thicknesses were critically examined and compared to infer the possible source area(s) for the sediments.

## 2 Stratigraphy

The Cape Supergroup is stratigraphically divided into the Table Mountain, Bokkeveld and Witteberg Groups (Figure 1) based on the age and depositional environments (Booth and Shone, 2005; Linol et al., 2016). The Table Mountain Group is the basal unit and it consists of sandstone, shale, conglomerate, siltstone and glacial diamictite. The Table Mountain Group (TMG) is subdivided into the Nardouw and Peninsula Subgroups (Thamm and Johnson, 2006). The Palaeozoic Table Mountain Group reaches an estimated maximum thickness of approximately 4430 m in the western side of the Cape basin and 4550 m in the eastern side; prominent outcrops with minimal duplication are present near Port Elizabeth town (Johnson et al., 2006). The quartz-rich sandstones of the Table Mountain Group metamorphosed to quartzites, forming the mountain ranges around Cape Town (Lubke and De Moor, 1998). The Bokkeveld Group is the middle unit of the Cape Supergroup. It overlies the Table Mountain Group and can be subdivided into the Ceres, Bidouw and Traka Subgroups (Johnson et al., 2006). The Devonian marine Bokkeveld Group displays five upward coarsening cycles with each been characterised by shales that grades into siltstones and sandstones at the top (Fourie, 2010). Based on the different sedimentation styles between the Table Mountain and Bokkeveld Groups, it is inferred that the basin must have been destabilised during the transition from Table Mountain to Bokkeveld sedimentation, thus resulting in transgression and regression (Fourie, 2010). The Late Devonian-Early Carboniferous Witteberg Group marks the end of deposition in the Cape Supergroup (Gess, 2013), and it can be subdivided into the Weltevrede Subgroup, Witpoort Formation, Lake Mentz Subgroup and Kommadagga Subgroup (Thamm and Johnson, 2006). The group attains a maximum thickness of about 1700 m in the western part of the basin, while it has been reported that the basin reached a maximum thickness of approximately 2600 m in the eastern part (SACS, 1980). The arenaceous Witteberg Group conformably overlies the argillaceous strata of the Bokkeveld Group and it is made up of shales, siltstones and quartzites.

The overlying Karoo Supergroup is stratigraphically subdivided into five major groups, namely the Dywka, Ecca, Beaufort, Stormberg and Drakenberg Groups (Johnson et al., 2006, Figure 1). The Dwyka Group is the lower most units of the Karoo Supergroup and it is envisaged that the group was deposited from the Middle Carboniferous up to the Early Permian (Linol et al., 2016). The Dwyka Group consists predominantly of massive diamictite of glacial origin and subordinate varved deglaciation shale and sandstone units, reaching a maximum thickness of around 800 m (Toerien and Hill, 1989; Visser; 1989; Linol et al., 2016). Deposition of the Dwyka Group marks the first deposition in the Karoo Supergroup and the deposition of the diamictites followed after the development of a shallow sea that resulted from deglaciation (Geel et al., 2015). The Ecca Group is marked by the lowermost black, organic-rich shales directly above the Dwyka Group. Linol et al. (2016) pointed out that the lowermost shale of the Ecca Group represents short-lived marine environment that resulted from rapid deglaciation of ice cap during Late Devonian to Middle Carboniferous. The middle to upper most units of the Ecca Group are more arenaceous, thicker and reflects transition into fluvial environments (Linol et al., 2016; Visser, 1989). These units consist of dark-grey silty shales intercalated with fine grained sandstone and carbonaceous mudstones (Visser, 1989). In the southern part of the basin, these deposits reflect outer fan turbidites that grades into shallow shelf slope sediments towards the north (Linol et al., 2016). The Beaufort Group conformably overlies the Ecca Group and it comprises of sandstone, shale, mudstone and siltstone. Johnson et al. (2006) subdivided the Beaufort Group into the Adelaide and Tarkastad Subgroups. The Adelaide Subgroup is the basal unit and it also crops out in the western portion of the main Karoo Basin and it is represented by Abrahamskraal and Teekloof Formations. The overlying Tarkastad Subgroup comprises of the Koonap, Middleton and Balfour Formations in the eastern part of the basin. The Middle Triassic Stormberg Group overlies the Beaufort Group and in turn overlain by the younger Drakensberg Group, which is the last set of rocks to be deposited in the Karoo Supergroup. The Drakensberg Group is believed to have formed during the Late Triassic - Early Jurassic period (183 Ma) through extrusion of magma along fissures and cracks (Drakensberg lava), thus capping the Karoo sedimentary rock with an approximately 1400 m thick layer of basalts (Van der Merwe et al., 2009).

## 3 Materials and methods

### 3.1 Dry density measurement

**Figure 2:**
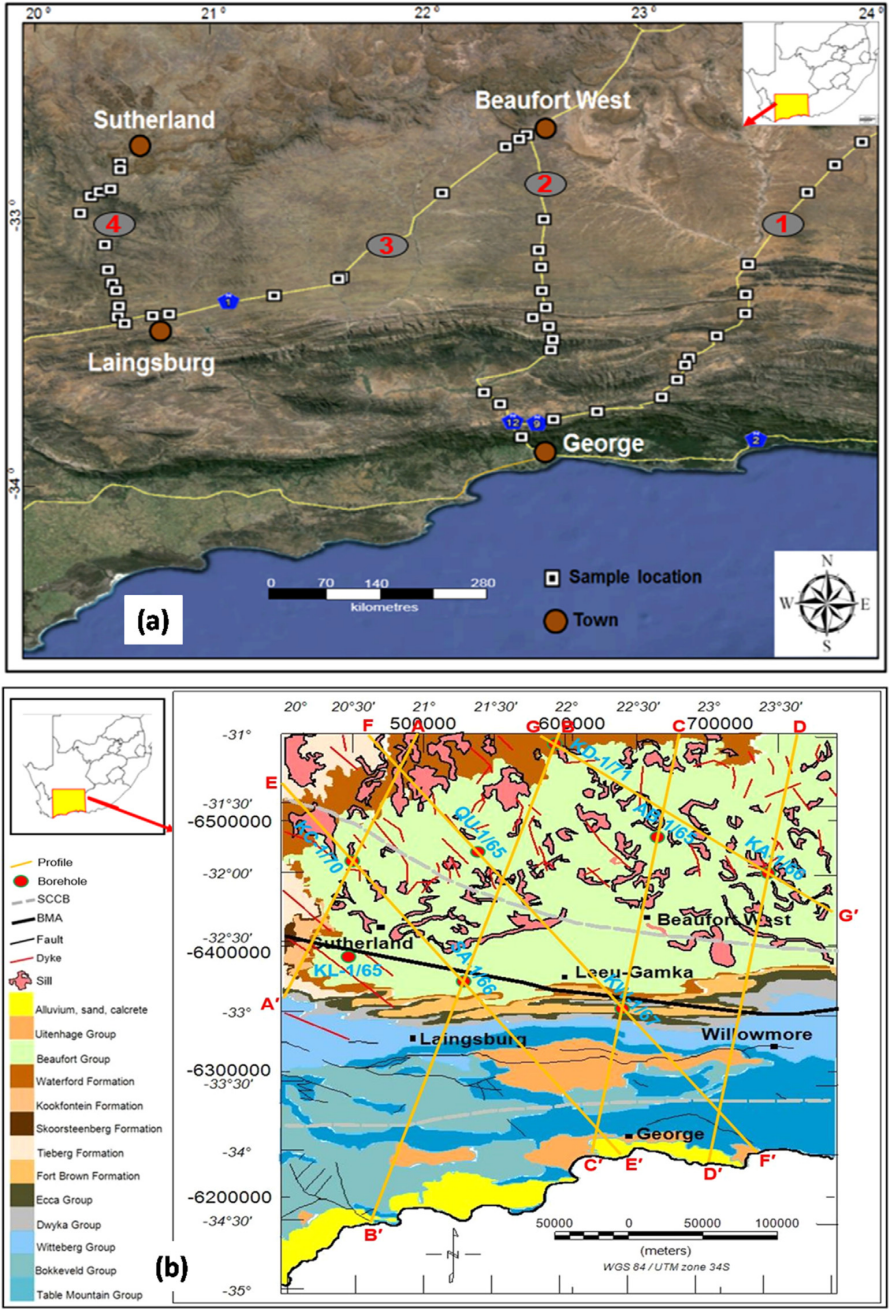
(a) An aerial photograph displaying the location of studied rock outcrops; (b) Geological map showing the location of boreholes and the major geophysical anomalies within the study area. Note: The grey and blue circles in Figure 2a indicate section numbers and names of national roads, respectively.

Seventy-one fresh rock samples (Figure 2) were collected from the outcrops of the Cape and Karoo Supergroups within a time period of six days. The collected samples are diamictite, shale, sandstone, mudstone, quartzite, limestone, chert, schist, and Pre-Cape conglomerate. The dry density of these rock samples were determined in the laboratory using Archimedes’ principle (Eq. (1)). The samples were dried in the sun for 3 days. Thereafter, the mass of each of the rock sample was weighed in air (M_d_) and when immediately submerged (M_b_) in water using an Adam PGW 4520e digital balance. In addition, the density of water was determined hourly during the mass measurements of the rock samples. A 30 ml density bottle was used and an average water density $\left({\text{ρ}}_{\text{w}}\right)$ of 1.017 g/cm^3^ was obtained.

(1) $$\text{Dry density }\left({\text{ρ}}_{\text{d}}\right)=\left(\frac{M_{d}}{M_{d-}M_{b}}\right)\times {\text{ρ}}_{\text{w}}$$

### 3.2 Gravity data acquisition and processing

The gravity data consisting of 12309 gravity stations was obtained from the Council for Geoscience in the form of xyz data file. The spacing between individual station is approximately 1–3 km apart. The gravity data had been reduced to Bouguer gravity values. The Bouguer gravity values are presented in the form of geophysical maps. As indicated by the Council for Geoscience, the drift, latitude, atmosphere, free-air and Bouguer corrections were applied to the original gravitational data. More detailed description on the gravity data reduction can be found in Telford et al. (1990). The reductions were applied using the Geosoft Oasis Montaj Gravity and Terrain Correction Module.

### 3.3 Profile selection and 2½ D gravity models

Seven gravity profiles (A-G) were systematically selected to cover the study area (Figure 2b). These profiles were modelled by extracting the Bouguer gravity and elevation values from several gravity station points along the selected profiles into an excel file and then imported to a GM-SYS software. GM-SYS is an extension on Geosoft Oasis Montaj which provides an interface for interactive manipulation of geological bodies (depth and location), and it performs real time calculations of gravity field response, thus enabling the calculated gravity field curve to match the observed gravity curve with a minimum root mean square error. The working principles of GMSYS and modelling procedures can be found in Baiyegunhi (2015) and Baiyegunhi and Gwavava (2016, 2017). GM-SYS version 7.3 (18) was used to produce forward models from the gravity profiles that traverses the study area (Figure 2b). Initially, seven gravity profiles were selected and positioned in such a way that they cut across the rock strata and geological structures perpendicular or near perpendicular (45–90°) to the strike direction across the study area. Six of the gravity profiles were orientated in such a way that they cut across the BMA and SCCB throughout the study area. The gravity profiles position and extent on the ground were determined from the geological map of the South Africa. Four profiles were oriented in a NE-SW direction, while the other three were positioned in a NNW-SSE direction nearly perpendicular to E-W dyke trends. The outcrop/thicknesses of various groups was extracted from the geological map of Southern Africa (Visser, 1989). The measured densities of both the Cape and Karoo Supergroup rocks were used in the modelling together with those from Baiyegunhi (2015). Also, the geological map of the study area was used to measure the actual profile lengths and positions along the profiles to each geologic group outcrop within the study area (Figure 2b) and at infinity (outside the study area but along the profile). This was done in order to set constraints to the model such that the model will perfectly fit or agree with what is obtained in the real-world.

A geologic model for a selected gravity profile was created and real-time calculation of the gravity response of a specific Earth model was performed. A starting GM-SYS model was created from a map profile (i.e. A - A’) by importing the gravity and elevation grids. The estimated thickness (length) and position of each group from the geological map were used to develop the starting model. Each starting model was extended to infinity at both ends (i.e., a long distance say ± 30,000 km in this case) to eliminate edge-effects. A constant or DC shift was assigned and subtracted from the calculated gravity data in order for the calculated gravity data (black line in Figures 5 and 6) to match the observed data (black dots in Figures 5 and 6). This was done because the calculated value is an absolute gravity calculation of the model extending to 30,000 km in the ±X directions. Geologic boundaries that cross the selected profiles were located in the starting models using the control points (red points) with respect to the measurements. These control points remain fixed while other points are moved or adjusted. The model was prepared for inversion by assigning names, densities, dimensions (length of blocks in +Y and –Y strike direction) to the blocks representing the various rock units. After preparing the model for inversion, the actual inversion process was initiated such that the calculated curves were updated in the anomaly panes, leaving dotted lines as the previous calculated curve which automatically changes the original model when accepted. The inversion process was repeated until the final model had an acceptable minimum root mean square (RMS) error (red line) between the calculated and observed gravity. The final model was inspected to see if it was geologically reasonable. The models were constrained using the densities values, stratigraphic thickness from outcrops and SOEKOR borehole as well as seismic refraction and magnetotelluric images provided in Stankiewicz et al. (2008) and Linol et al. (2016). The Moho depth have been indicated to vary between 40–45 km below the sea level (Baiyegunhi and Gwavava, 2017; Stankiewicz and de Wit, 2013). To cover the reported depths for the Moho, the depth to the Moho was initially set at 40 km and allowed to vary up to 45 km during the modelling process. This will enable model to establish the best fit depth for the Moho. The thickness of the modelled dolerite intrusions was estimated from Chevallier et al. (2001) and Chevallier and Woodford (1999). The modelled dolerite sills and dykes were originally set in horizontal and vertical positions, respectively and allowed to vary during the modelling process to have the best fit model. For each profile, 3 models were generated using the minimum, average and maximum density values. Since a total of 21 models were obtained, only those of the average density values are presented in this paper. The alternative models were used to check the sensitivity of the models with respect to change in layer densities and thicknesses.

### 3.4 Estimation of isochore (vertical) thicknesses

The elevation and isochore thickness maps were both constructed using the depth/elevation data of various geological groups obtained from the GM-SYS gravity models relative to their position above or below sea-level. The following steps were executed to construct depositional surface and thickness maps for the various groups:

1. The top layer of the Cape Supergroup was exported in real world xyz file system from the GM-SYS model to an excel spreadsheet, where z is an elevation at a particular point along the profile. This was done for all the profiles (Profile A-A′ to G-G′) and the elevation data for the Cape Supergroup top layer was compiled into a single excel sheet.
2. The created spreadsheet with Cape Supergroup top layer was then imported into Geosoft Oasis Montaj database, where it was gridded and displayed as Cape Supergroup elevation map.
3. The above procedure (i.e. step 1 and 2) was executed for the top layers of the Dwyka, Ecca and Beaufort Groups.

It was then deduced that the top of the basement equals the bottom layer of the Cape Supergroup (Eq. (2)), the top layer of the Cape Supergroup corresponds to the bottom layer of the Dwyka Group (Eq. (3)), top layer of the Dwyka Group corresponds to the bottom layer of the Ecca Group (Eq. (4)), top layer of the Ecca Group equals the bottom layer of the Beaufort Group (Eq. (5)) and this applies to the top of Beaufort Group (Eq. (6)), provided the principle of superposition is preserved. This can be expressed mathematically as:

(2) • Basement top = Cape Supergroup bottom

(3) • Cape Supergroup top = Dwyka Group bottom

(4) • Dwyka Group top = Ecca Group bottom

(5) • Ecca Group top = Beaufort Group bottom

(6) • Beaufort Group top = Beaufort Group top

It was further deduced from the above mathematical expressions that, if the top surface grid of a particular geological group (Eqs. (7), (8), (9), (10)) is subtracted from the bottom surface grid of the same group (Eqs. (7), (8), (9), (10)), the result would be the isochore thickness grid of that particular geological group. The latter statement can be expressed as:

(7) • Cape Supergroup thickness = Cape Supergroup top−Cape Supergroup bottom

(8) • Dwyka Group thickness = Dwyka Group top−Dwyka Group bottom

(9) • Ecca Group thickness = Ecca Group top−Ecca Group bottom

(10) • Beaufort Group thickness = Beaufort Group top−Beaufort Group bottom

The above mathematical expressions were used to estimate the isochore thicknesses of the various geological groups. For example, the top surface grid of Ecca Group was subtracted from the bottom surface grid of Ecca Group using grid math in Geosoft Oasis Montaj and the resulting grid represents the isochore thickness of Ecca Group. This was done for all the geological groups and the grids were presented as isochore thickness maps. The deduced isochore thickness maps were compared with the seismic and borehole results to test their accuracy. For each of the elevation grids, the average value was subtracted in order to reduce each surface relative to the sea level. In addition, the elevations (i.e. top surface of each geological group) are the remaining elevation after subsidence, deformation, and physical processes acted on the geologic sequence during and after deposition. The elevation for the geologic groups were recovered by extracting each surface (horizon) for all the profiles in xyz coordinates. More detailed about the modelling procedures and thickness estimation can be found in Baiyegunhi and Gwavava (2016, 2017).

## 4 Results and discussion

### 4.1 Density

The measured density for the selected rocks are presented in Table 1. The dry density of the rocks varies between 2.2 - 2.7 g/cm^3^ and they follow no specific pattern with reference to the stratigraphic sequence. Instead, they vary within and from one group to another. Most of the groups that are enriched in shale and mudstone have the lowest densities, with a slightly higher porosity as compared to those of quartzite and sandstone. Similar to the findings of Allen and Allen (2013), and Punmia et al. (2005), the density of studied rocks increases with stratigraphic depth. This can be seen on the rocks of the Karoo Supergroup, which shows a slightly lower density than the underlying Cape Supergroup. This might be due to the fact that the Cape Supergroup rocks have undergone recrystallization as well as isothermal compression.

**Table 1:** The minimum, maximum and average dry densities used in the gravity modelling.

Supergroup	Group	Min density (g/cm^3^)	Max density (g/cm^3^)	Avg density (g/cm^3^)
Cenozoic deposits		2.200	2.616	2.408
	Uitenhage	2.435	2.536	2.513
Karoo dolerite		2.579	2.816	2.697
Karoo Supergroup				
	Beaufort	2.457	2.499	2.478
	Ecca	2.315	2.675	2.497
	Dwyka	2.301	2.750	2.535
Cape Supergroup		2.549	2.744	2.665
Pre Cape (basement)		2.673	2.730	2.702
Mantle		3.140	3.400	3.270
Min = Minimum		Max = Maximum		Avg = Average

The table above presents min, max and average densities of various rock groups that were measured in the laboratory together with those that were extracted from the literature. Note: The outcrops of Cenozoic, Uitenhage and Mantle rocks were not accessible in the field. Therefore for the purpose of modelling their densities were extracted from Baiyegunhi (2015) and Allen and Allen (2013).

### 4.2 Gravity

**Figure 3:**
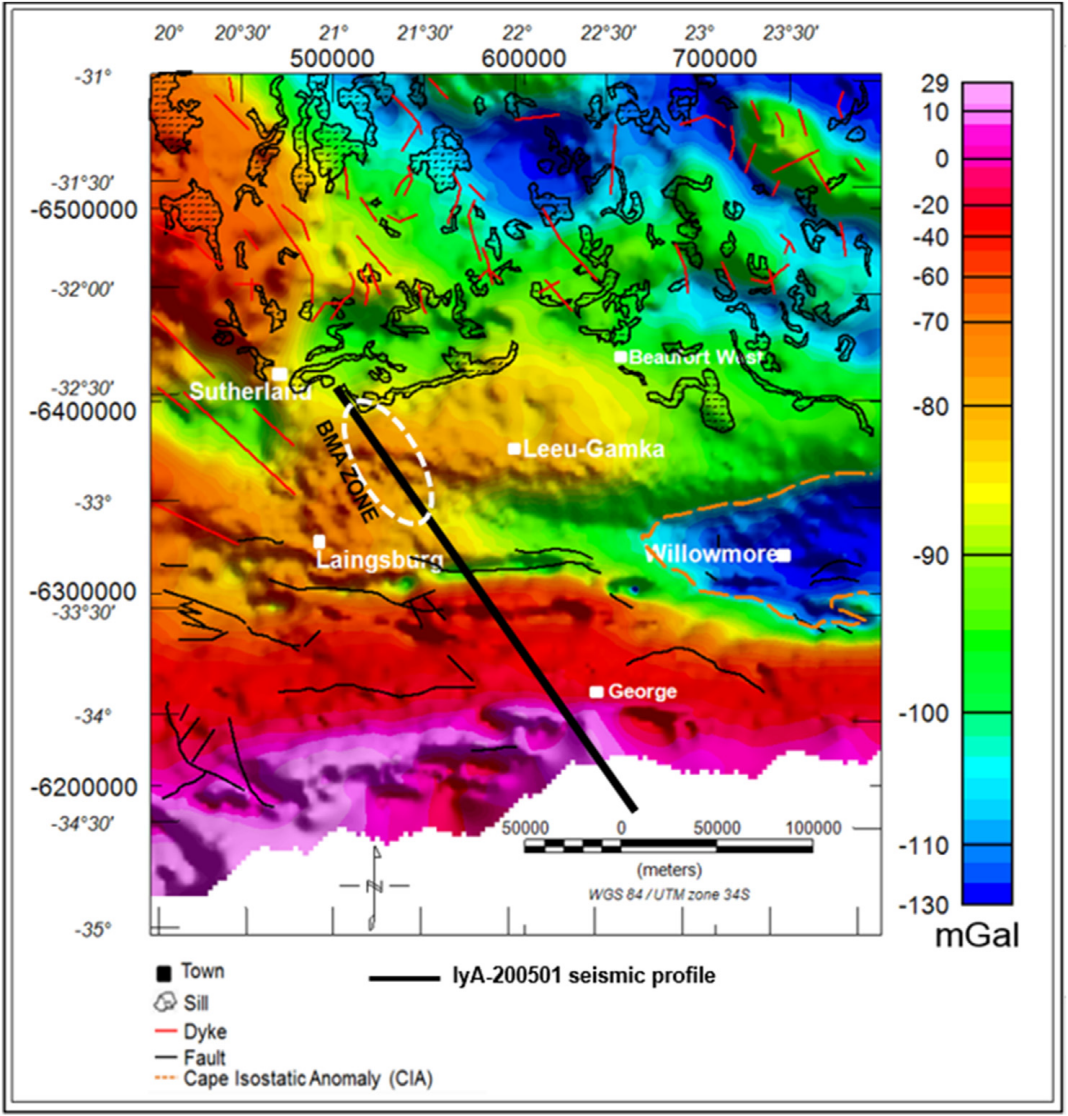
Bouguer gravity map overlain on the geology.

**Figure 4:**
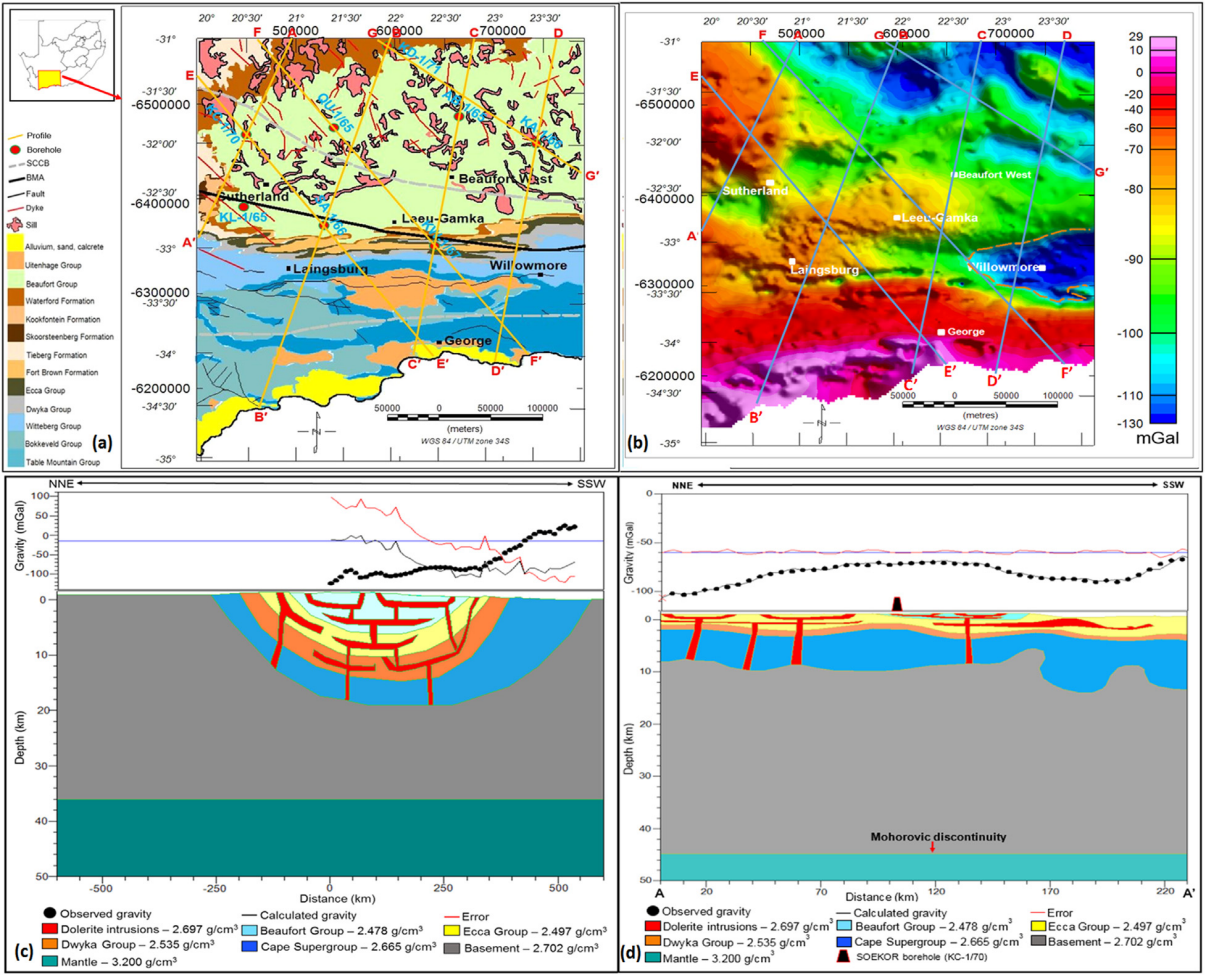
(a) The southwestern Karoo Basin geological map showing the location of gravity profiles and eight SOEKOR boreholes within the study area; (b) Bouguer gravity map of the southwestern Karoo Basin.; (c) A geological representation of subsurface starting model; (d) A geological subsurface model along profile A-A'. Vertical Exaggeration (VE) = 3.02. Initial and final Root Mean Square (RMS) error are 87.6 and 1.7, respectively. Note: The Moho was allowed to vary within a depth range of 40–45 km. At borehole KC-1/70, 105 km in SW direction, the base of the Dwyka Group is encountered at 1745 m below the surface.

**Figure 5:**
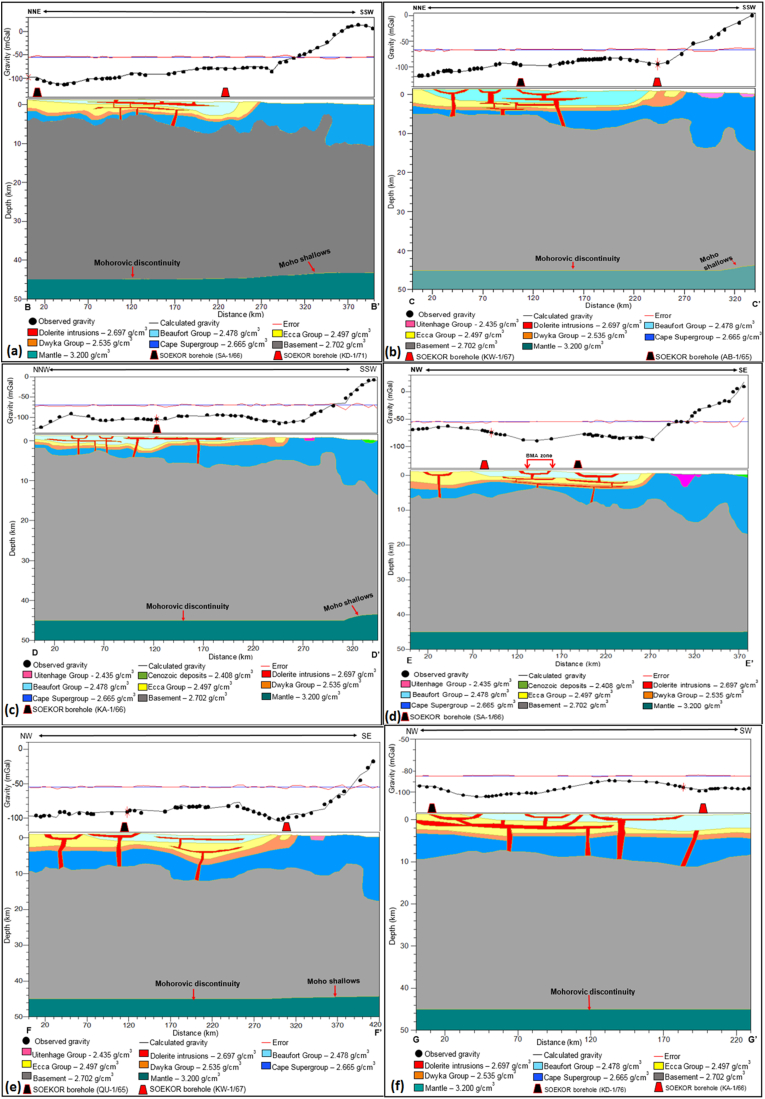
Gravity modelling of profiles: (a) B-B’; (b) C-C’; (c) D-D’; (d) E-E’; (e) F-F’ and (f) G-G’. Note that when the black line/curve (calculated gravity) matches with the black dots (observed gravity), it indicates a perfect fit with minimum error.

The Bouguer gravity map shows several significant zones of anomalous highs and lows within the study area (Figure 3). The gravity values vary from -130 mGal up to 29 mGal. Some of the gravity anomalies does not correlate with any mapped geology on the surface. Hence, they are likely to be caused by subsurface sources. Towards the coast, in the far south, a 50 km wide east-west trending gravity high coincides with the Cape Fold Belt. This signature continues along the Cape Fold Belt towards the east of the study area for several kilometres. The dominant gravity variation is of long wavelength and it is possibly due to a deeper source/interface inland that shallows toward the coast. This is likely to be the effect of the steeply rising Moho near the coast as shown on the model depicted in Figures 4 and 5.

### 4.3 2½ gravity profile modelling

The gravity modelling result of profile A–A’ is shown in Figure 4. Profile A-A′ extends for approximately 230 km in a NNE-SSW direction and ends about 50 km south of Sutherland town. Along profile A-A′, the gravity reaches a maximum and minimum of about -64 mGal and -102 mGal, respectively. It is evident from the model that the geological formations are sub-parallel to each other with folding, faulting, and intrusions in places. An interpretation of the subsurface model, from the oldest geological formation to youngest, reveals that the Cape Supergroup extends to the depths of approximately 12 km below sea level in the south, with the top surface occurring approximately at 2 km below sea level in the north. The Dwyka and Ecca Groups occur between the depths of 0–3 km and both groups show a lateral uniform thickness along the profile with gentle folds in places, while the overlying Beaufort Group occurs above the sea level.

**Figure 6:**
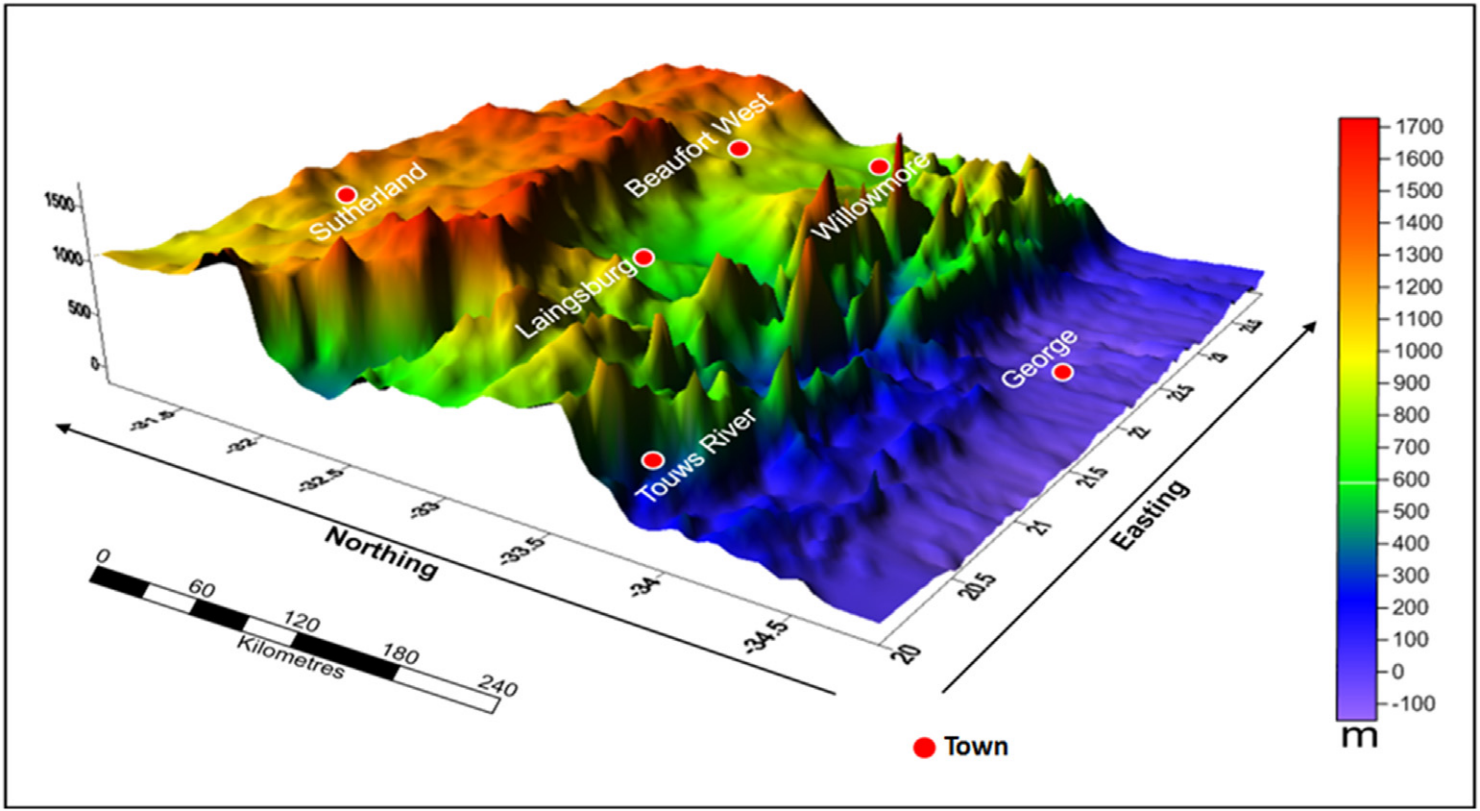
A 3D representation of the current elevation in the study area showing topographic highs and lows in the main basin. The red coloured zones are high elevation areas, while areas marked by blue colour represent low elevation zones.

The thickness of sediments in the Karoo and Cape Supergroups varies along profiles B – G as a result of deformation (Figure 5). The sedimentary sequence of the Karoo Basin is intruded by dolerites with associated faulting as depicted in Figure 5. These Karoo dolerites occur as a network of interconnected dykes and sills with some having the same parent feeder dyke. The intrusions, mostly the sills, occurred as basin like structures (ring/saucer), while the dykes are commonly associated with the faults. The dykes acted as feeders to the sills that are outcropping on the surface. The structure of the dolerite intrusions in Figures 5 and 6 is similar to those of Chevallier et al. (2001), and Baiyegunhi and Gwavava (2017). In addition, the thickness/depth depicted in the gravity models (Figures 4 and 5) are also consistent or similar in values with the borehole data (i.e. boreholes AB- 1/65, KA-1/66, KC-1/70, KD-1/71, KL-1/65, KW-1/67, QU-1/65 and SA-1/66) as well as seismic refraction and magnetotelluric models of Weckmann et al. (2007), Lindeque et al., (2011) and Scheiber-Enslin et al. (2015). The aforementioned models show that the Karoo rocks occur at deep depths in the south and become relatively shallow towards the north. This could be attributed to the prominent tectonic deformation in the southern part of the basin. Roberts and Bally (2012) described a series of subsidence and regional uplift events that are likely to have shaped the tectonic setting of the Karoo and Cape Basins. Also, the illustration of dolerite intrusions shown by the models is similar to the model of Chevallier et al. (2001), which shows a network of dolerite intrusions in the subsurface slicing through the Cape and Karoo sediments. These interconnected dolerite networks could pose a serious threat to shale gas exploitation in the study area.

### 4.4 Depositional surfaces and isochore thicknesses of the Karoo Supergroup

The 3D image of the current elevation surface of the study area is shown in Figure 6. The topography displayed in Figure 6 is typical of the main Karoo Basin, which is characterised by flat, nearly smooth topography on the northern parts and abruptly changes into a basin like structure towards the south near Beaufort West and further changes into a rugged, hilly topography (Cape Mountains) further to the south.

#### 4.4.1 Elevation and isochore (true vertical) thickness maps of the Cape Supergroup

**Figure 7:**
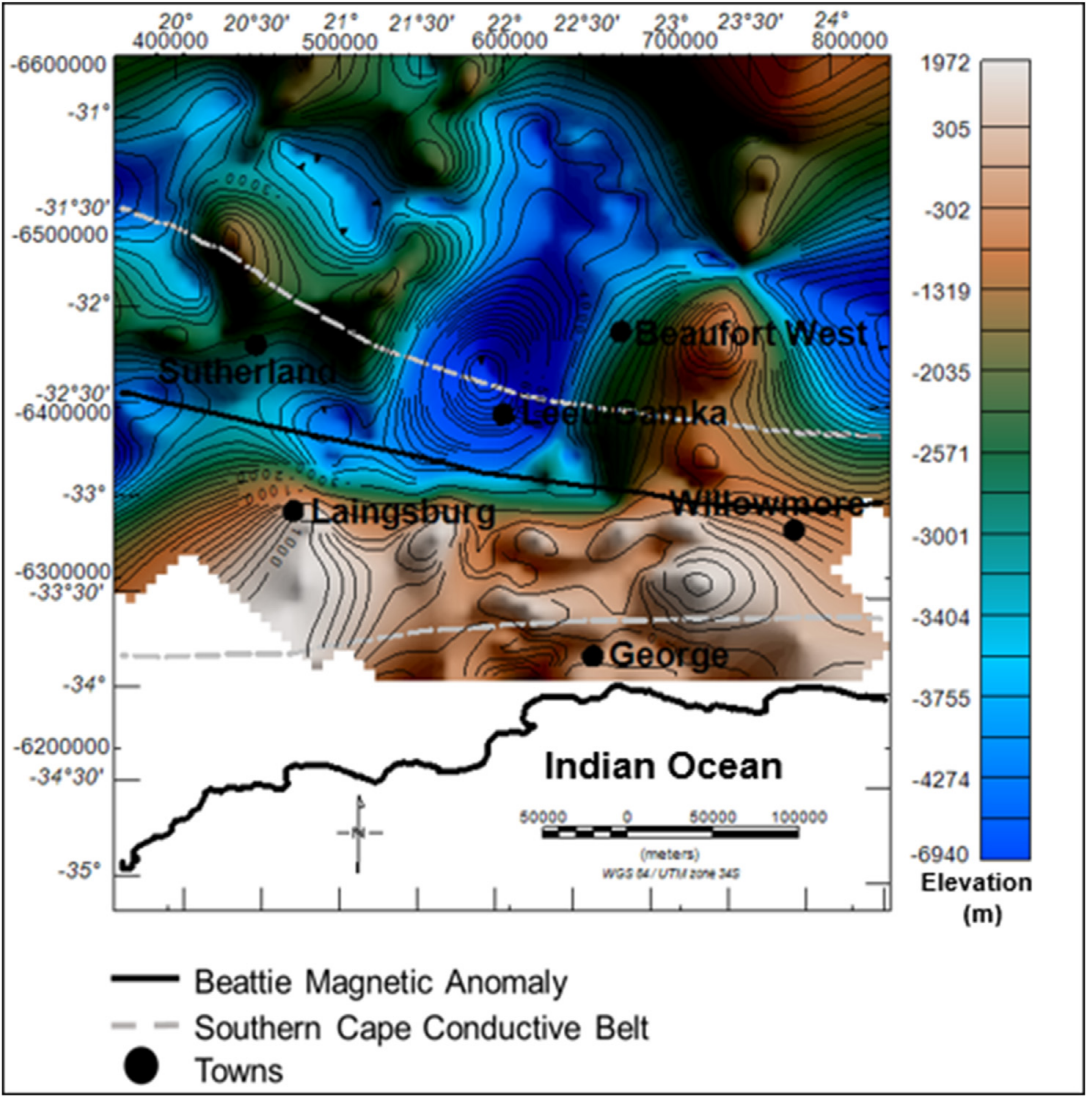
Generalised current elevation of the Cape Supergroup. Note: The topographic map represents the current elevation after deformation. The negative values denote elevation below the sea-level.

**Figure 8:**
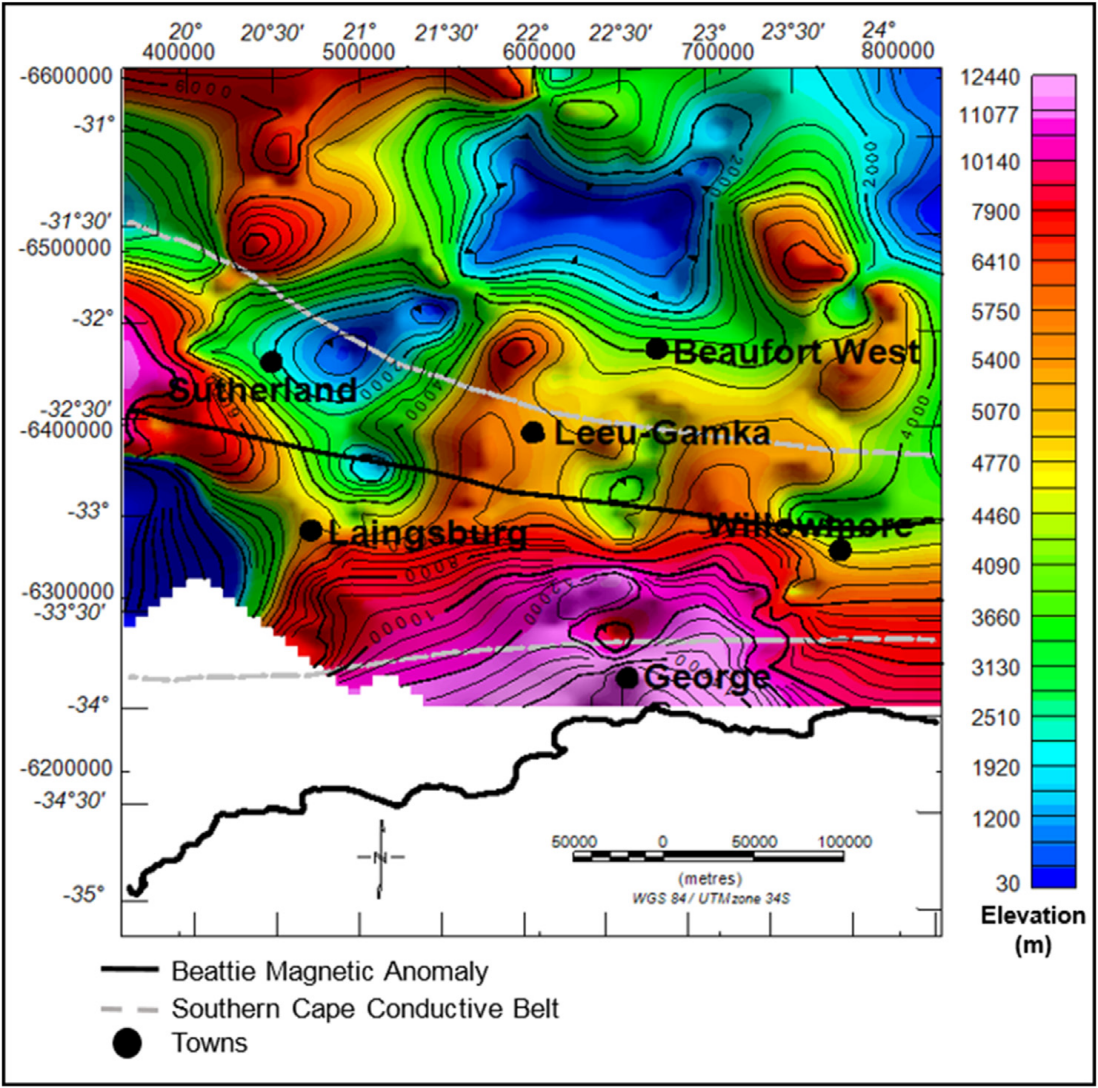
Vertical thickness map of the Cape Supergroup sedimentary rocks. The map shows variations in thickness of the Cape Supergroup within the study area.

The current elevation of the Cape Supergroup ranges from – 6940 m to 1972 m. The light-dark brown colour in Figure 7 represents structural highs while the green to blue-zones are the structural lows areas. The current elevation is the elevation after the rocks have been deformed. The Cape Supergroup thickness decreases from the southern part of the basin to the northern part of the basin, with a maximum thickness of greater than 12130 m in the south and a minimum of less than 100 m in the north (Figure 8). Scheiber-Enslin et al. (2015) reported that the Cape Supergroup pinches out in the northern side of the basin.

#### 4.4.2 Elevation *and isochore (true vertical) thickness maps of the Dwyka Group*

**Figure 9:**
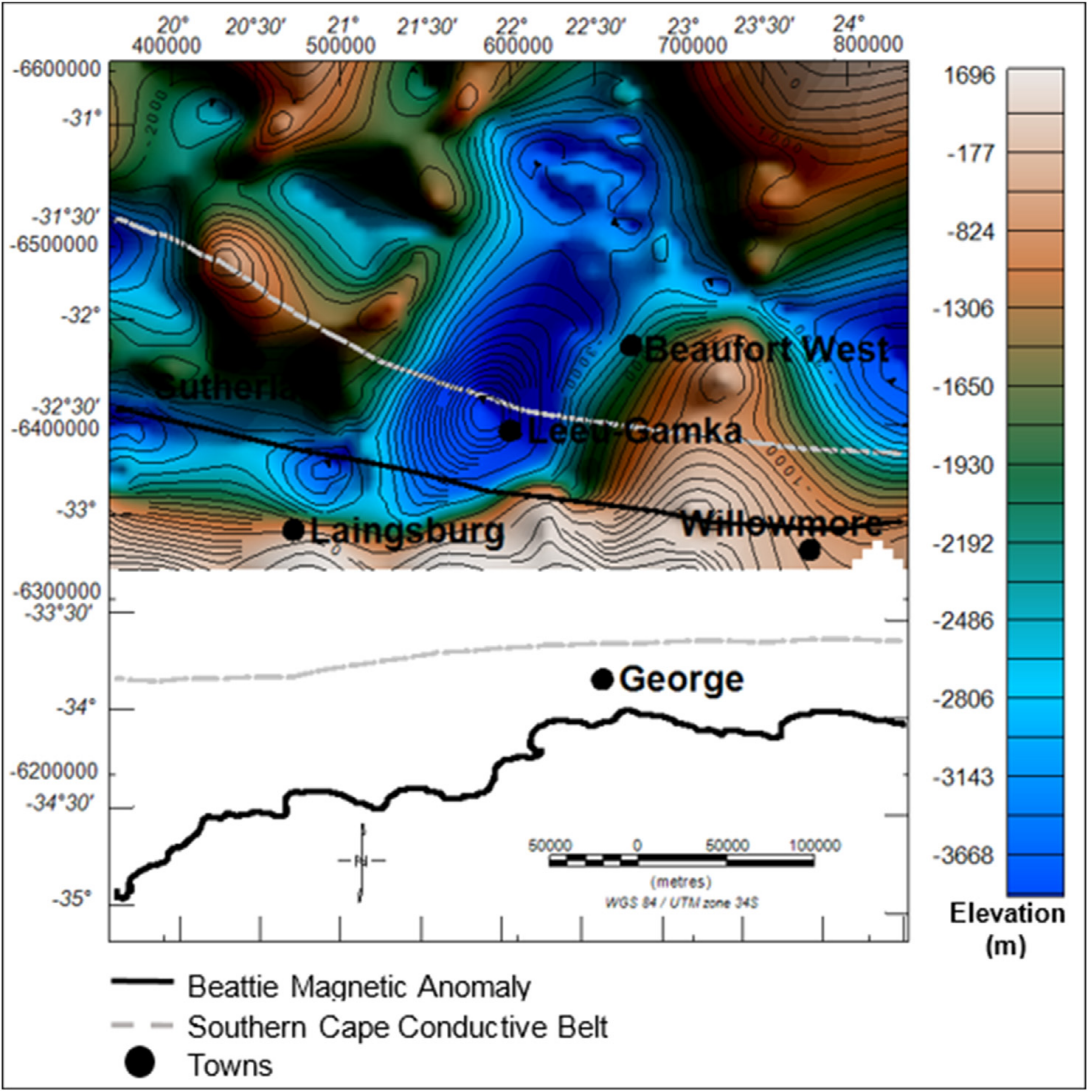
Generalized current elevation of the Dwyka Group. Note: The topographic map represents the current elevation after deformation. The negative values denote elevation below the sea-level.

**Figure 10:**
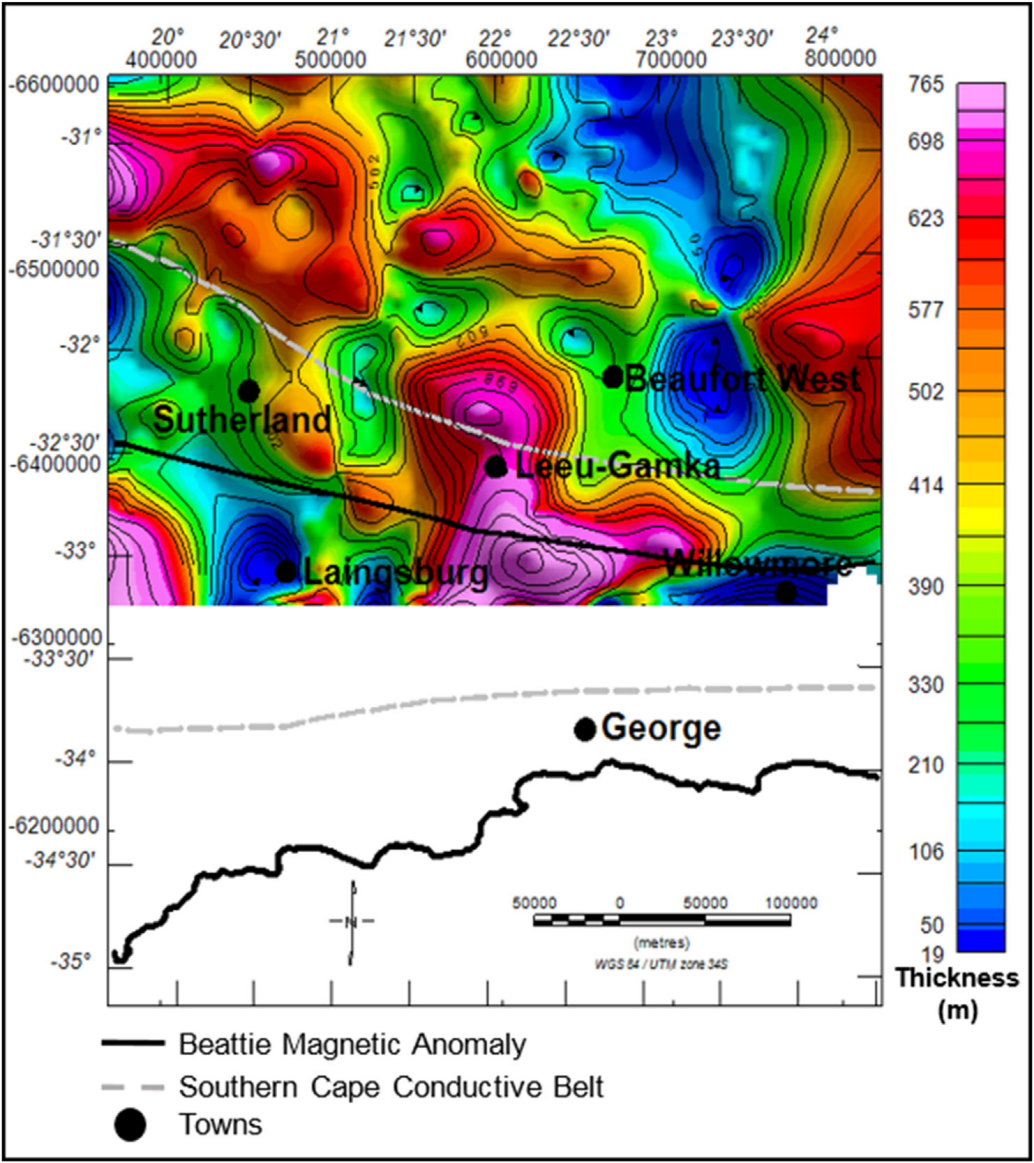
Vertical thickness map of the Dwyka Group sedimentary rocks. The map shows variations in thickness of the Dwyka Group within the study area.

The general topography of the Dwyka Group is characterised by both high (light-dark brown) and low (green-blue) relief areas (Figure 9). The highly elevated areas have been identified as the structural highs, with a vertical relief of -1306 m below sea-level and 1696 m above sea level. The low lying areas (structural lows) have a minimum of less than -3668 m to a maximum of -1650 m below the sea level. The structural low areas are more prevalent at the centre of the basin. It is evident in Figure 10 that the Dwyka Group is tectonically thickens in the southern part of the basin near Leeu-Gamka and north west of Sutherland and west of Laingsburg town. In these areas, the group has a relatively high thickness ranging between 1046 m and 1718 m.

#### 4.4.3 Elevation and isochore (true vertical) thickness maps of the Ecca Group

**Figure 11:**
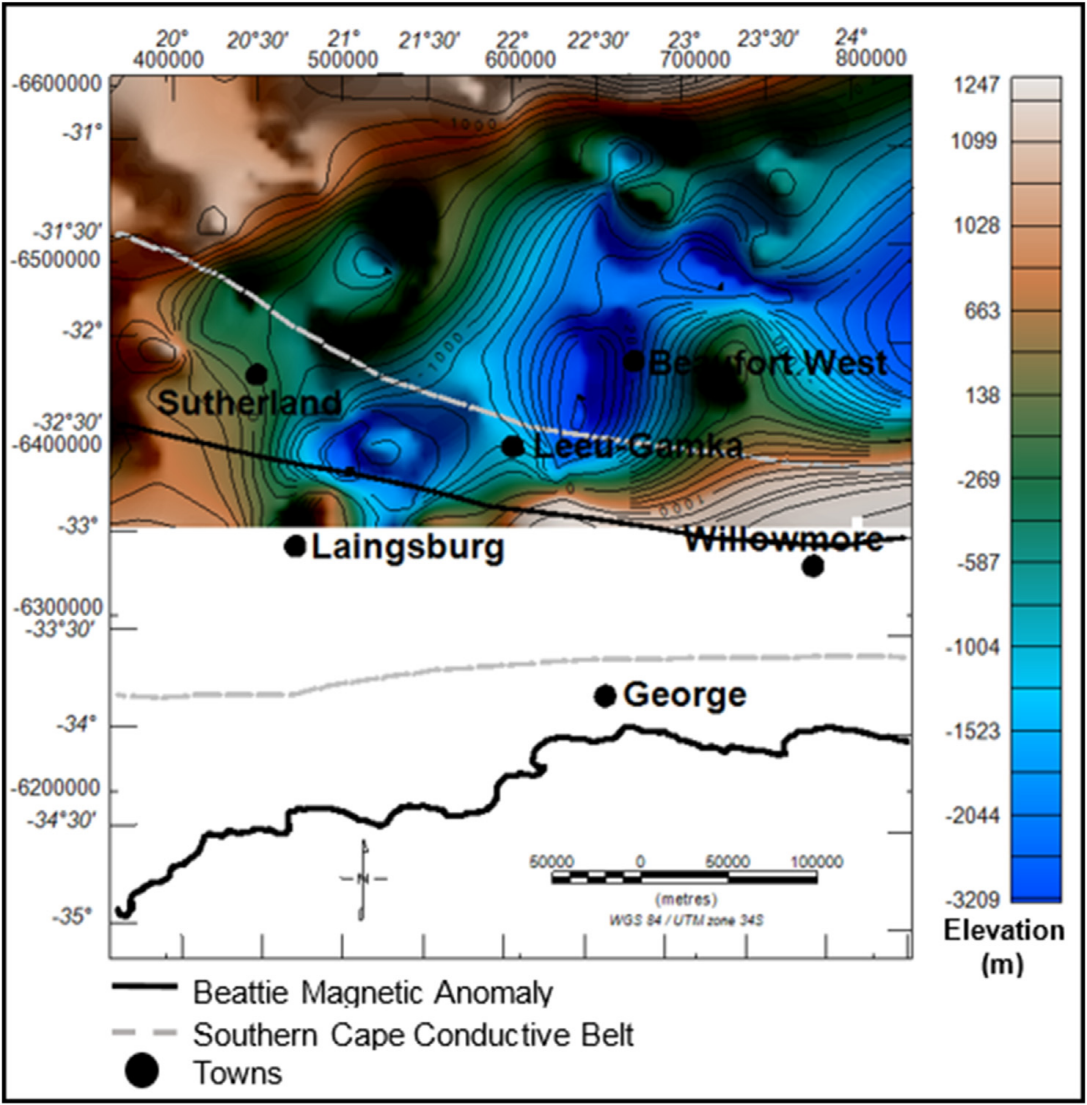
Generalised current elevation of the Ecca Group. Note: The topographic map represents the current elevation after deformation. The negative values denote elevation below the sea-level.

**Figure 12:**
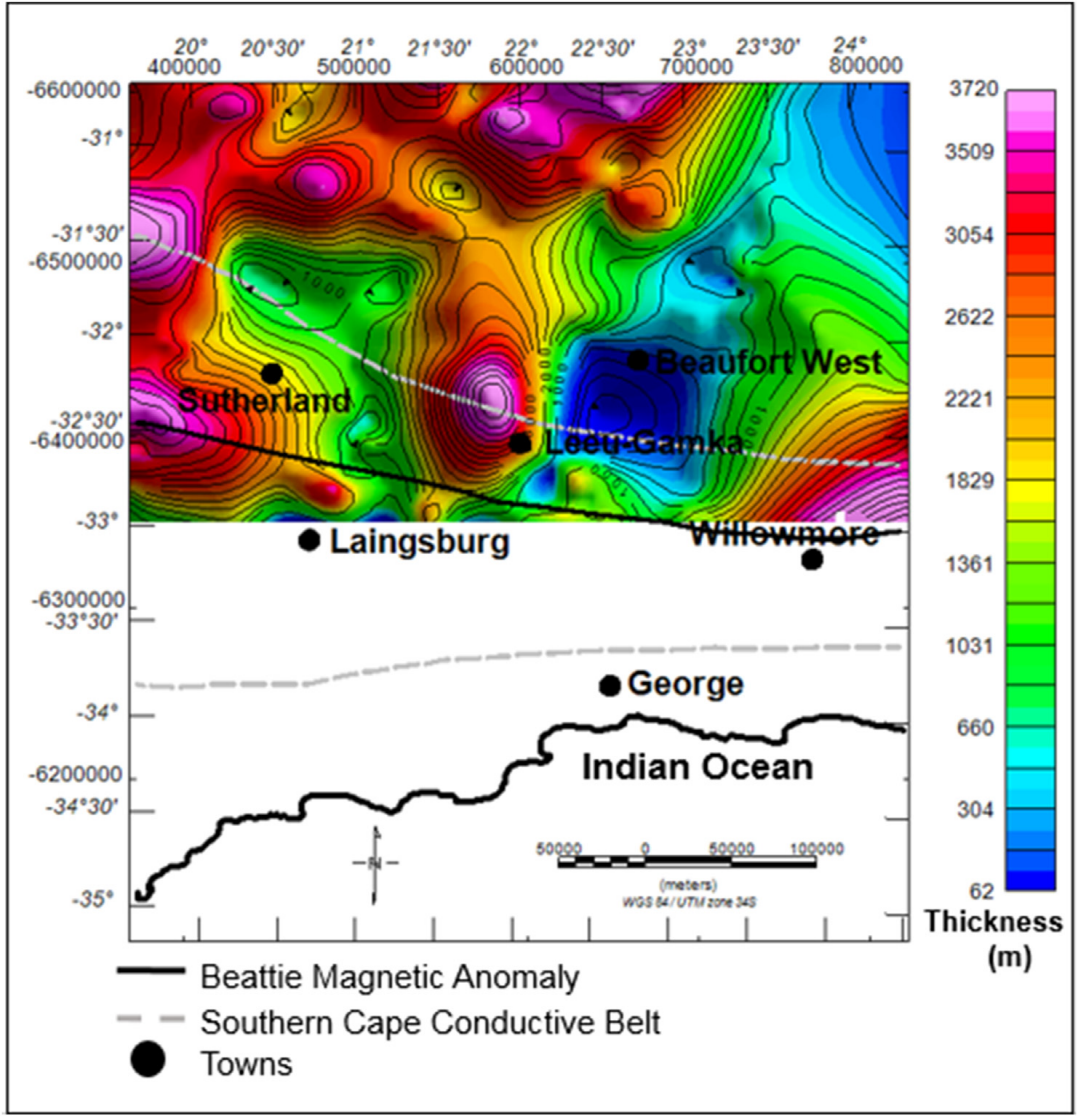
Vertical thickness map of the Ecca Group sedimentary rocks. The map shows thickness of Ecca Group succession in various parts of the basin.

The dark brown to light coloured zones in Figure 11 are structural highs with peak elevation of greater than 663 m, while the green-blue zones are structural lows with an elevation of less than -138 m (Figure 11). The Ecca Group shows a minimum and maximum elevation of -3209 and 1247 m, respectively. The Ecca Group reaches a maximum vertical thickness of more 3720 m in Figure 12. The succession shows a variable thickness throughout the basin, but it appears to be much thicker in the northern and north western part of the basin and thins at the centre and towards the eastern part of the basin (Figure 12).

#### 4.4.4 Elevation and isochore (true vertical) thickness maps of the Beaufort Group

**Figure 13:**
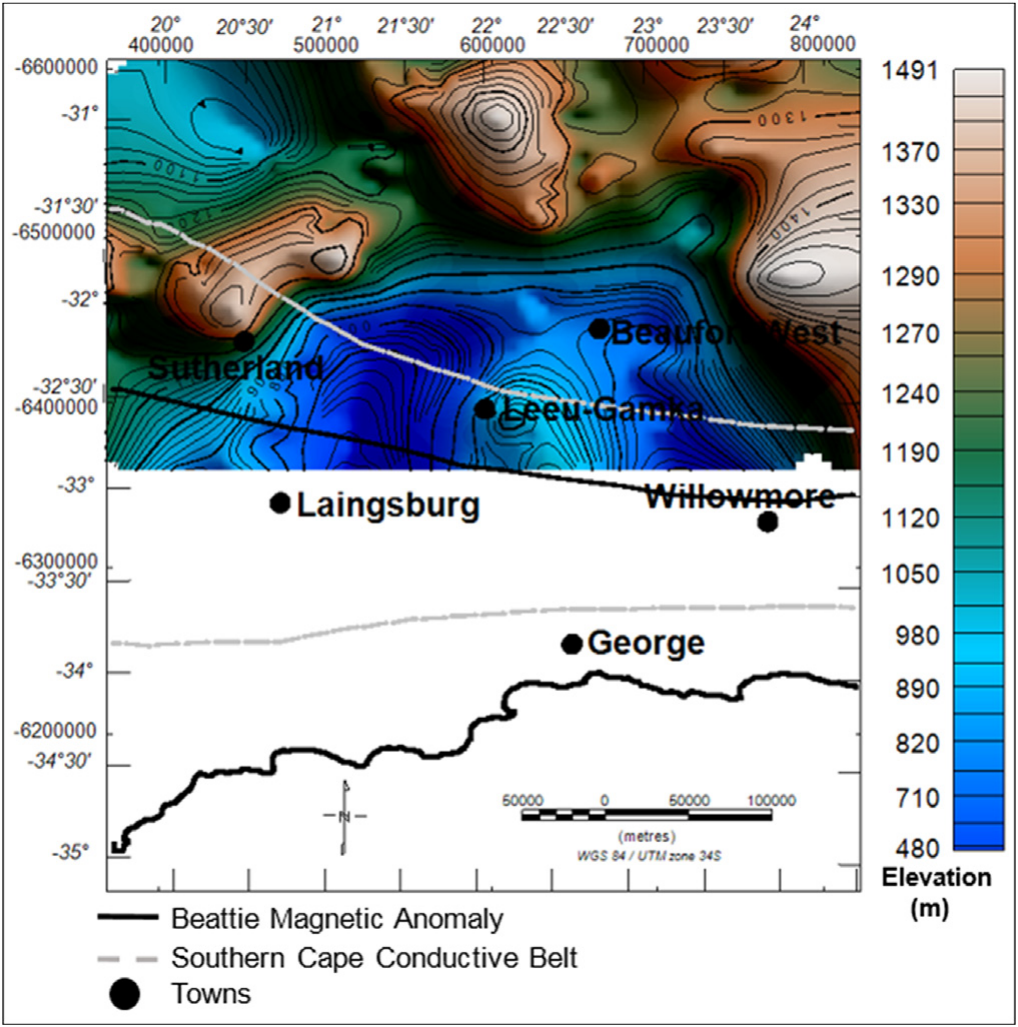
Generalised current elevation of the Beaufort Group. Note: The topographic map represents the current elevation after deformation. The negative values denote elevation below the sea-level.

**Figure 14:**
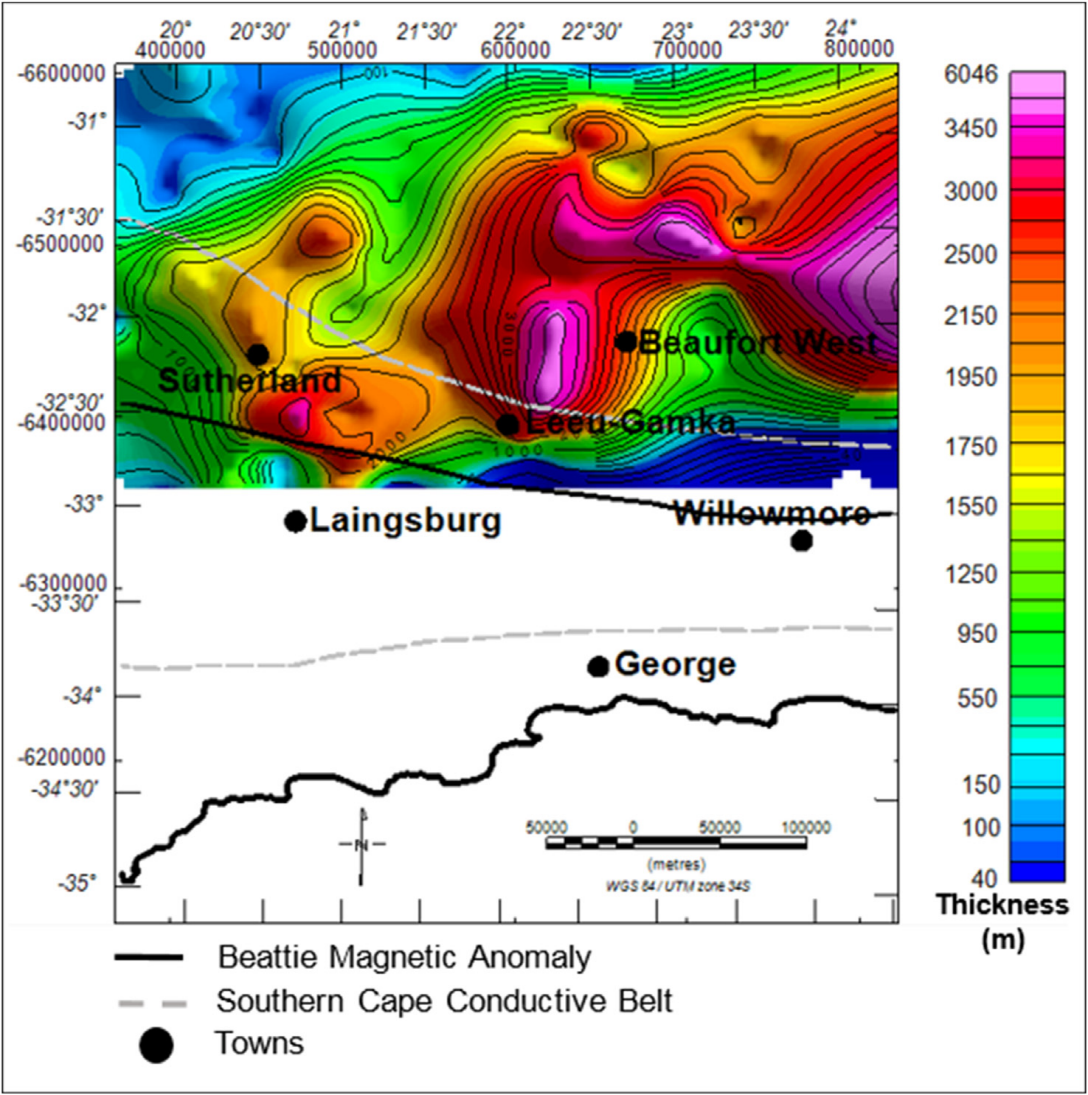
Vertical thickness map of the Beaufort Group sedimentary rocks.

The elevation of the Beaufort Group ranges from about 478 m to more than 1479 m, relative to the sea-level. The brown to light colour areas represent structural highs, while the green-blue colour represents the structural lows (Figure 13). The sedimentary rocks of the Beaufort Group appear to be more thick at the centre of the basin within the vicinity of the Beaufort West, Leeu-Gamka and Sutherland towns (Figure 14). In these localities, the basin is relatively deeper compared to other parts, particularly in the north where the topography is characterised by flat lying horizontal beds. The Beaufort Group reaches a maximum thickness of more than 3588 m in the northern side of Beaufort West, whereas towards the northern part of the basin, the strata become relatively thin to less than 100 m (Figure 14).

### 4.5 Sensitivity of the gravity models to change in density

Several or multiple gravity models were generated using the minimum, average and maximum density values along all profiles. This was done to determine variations in depth and isochore thicknesses as the densities of geological blocks changes. In addition, the sensitivity of the gravity models was also tested by comparing the variation in the derived isochore thicknesses at every grid point (198 ×102) in the model (i.e. total of about 20196 grid points), as the density values assigned to the blocks (groups/supergroup) changes from minimum density values, to average and finally to maximum the density values (Table 2). The comparison of maximum thicknesses for the Karoo Supergroup from different studies is presented in Table 3. The maximum vertical thicknesses obtained for each group (Karoo Supergroup) from the GM-SYS model are related to the maximum thicknesses calculated for the groups by other researchers like Johnson et al. (2006), Alao and Mikes (2011), and Baiyegunhi and Gwavava (2016). The slight differences in the thicknesses is presented in Table 2 could be due to the fact that the vertical thicknesses were obtained in this study rather than stratigraphic thicknesses.

**Table 2:** The sensitivity of the isochore thicknesses to density changes.

Group/Supergroup	Avg ρ(kg/m^3^)	Δ ρ (kg/m^3^)	Min ΔH (m)	Max ΔH (m)	Avg ΔH (m)	Error (m)
Beaufort	2478	+21	145	409	277	±277
		-21	144	408	276	
Ecca	2497	+178	78	286	182	±183
		-182	82	287	185	
Dwyka	2535	+215	11	123	67	±69
		-234	15	134	70	
Cape	2665	+109	96	247	172	±173
		-116	97	250	174	
Pre- Cape	2702	+28	25	98	61	±64
		-29	33	98	66	

Note: The Avg ρ is the average density value that was used to model every individual geological block and Δ ρ is the change in average density. The positive sign indicate an increase from average to a maximum density and is represented by the expression Δ  ρ= Maximum density - Avg ρ, while the negative sign denote the decrease and is expressed as Δ  ρ= Minimum density - Avg ρ. The Min ΔH and Max ΔH are the minimum and maximum change in isochore thickness due to density changes, respectively. Avg ΔH denotes average change in isochore thickness as the result of changes in density. The Error is the sensitivity of the model due to changes in the density.

**Table 3:** Comparison of maximum thicknesses of the Karoo Supergroup.

Group	This study	Baiyegunhi and Gwavava (2016)	Johnson et al. (2006)	Johnson (1976)
	Current isochore thickness	Isochore thickness (Southeastern Karoo Basin)	Stratigraphic thickness	Stratigraphic thickness
	Max. Thickness (m)	Max. Thickness (m)	Max. Thickness (m)	Max thickness (m)
Beaufort	6046±277	6342 ± 295	6850	6000
Ecca	3720±183	3207 ±263	3400	3000
Dwyka	765±69	727 ± 25	750	700

According to Tankard et al. (2012), the sedimentary fill of the Karoo Basin is due to crustal uplift, fault-controlled subsidence, and long periods of regional subsidence. Generally, the geology of the basin is characterised by regions of structural lows and highs, resulting in the thickness variations throughout the basin. Originally, the rocks were laid down horizontally in a varieties of depositional environments and were later affected by tectonism, thus causing folding and faulting as evident in Figures 4 and 5. The Karoo Basin responded to eight tectonic events in relation to the subduction of the palaeo-Pacific plate beneath Gondwana. These tectonic events produced variation in the depositional sedimentary successions within the Karoo setting (Catuneanu et al., 1998, Figures 10, 12, and 14). Based on the correlation of the isochore thickness and depositional surface (elevation) maps, it can be inferred that areas with low sediments accumulation are associated with structural highs (where erosion is more dominant). Conversely, areas with thick sediments cover are associated with structural lows with depression during their deposition, hence serving as sub-basin in which the eroded sediments were deposited. Furthermore, it is observed that the sedimentary cover is thick in the western part and towards the north-eastern parts of the study area. Therefore, these areas are likely to favour hydrocarbon accumulation when other conditions necessary for hydrocarbon development are present.

## 5 Conclusions

This study has shown that the applications of geophysical surveys combined with geological investigations provide a powerful tool in unravelling the configuration of the south-western Karoo Basin and the isochore thicknesses of the various geologic groups. The gravity modelling results show that the basin extend to the depth of 4500 m in the south, near the Cape Fold Belt and extend to shallows depths of 2600 m in the north. In addition, the models revealed that the Karoo dolerites are interconnected at depths and are mostly concentrated at the centre part of the study area. The isochore thickness maps indicate that the Ecca Group, which is the main target for hydrocarbon exploration, thickens away from the centre of the basin and reaches a maximum thickness of about 3680 m in the southern part of the study area. The Beaufort Group is the thickest succession in the Karoo Basin with a maximum thickness of about 6046 m. The correlation of the depositional surfaces with the thickness maps revealed that the sediments in structural high areas were subsided, eroded and deposited in the structural low areas. Hence, the structural low areas are characterised with thick sediments cover and vice versa. This study will possibly serve as a basis for future basin evolutionary models of the main Karoo Basin.

## Declarations

### Author contribution statement

Zusakhe Nxantsiya: Performed the experiments; Analyzed and interpreted the data; Contributed reagents, materials, analysis tools or data; Wrote the paper.

Oswald Gwavava: Conceived and designed the experiments; Analyzed and interpreted the data; Contributed reagents, materials, analysis tools or data.

Christopher Baiyegunhi: Performed the experiments; Contributed reagents, materials, analysis tools or data; Wrote the paper.

### Funding statement

Thanks to the Council for Geoscience for providing sponsorship/financial support received by Mr Nxantsiya.

### Data availability statement

Data will be made available on request.

### Declaration of interests statement

The authors declare no conflict of interest.

### Additional information

No additional information is available for this paper.
